# Effect of interleukin-6, -17, -21, -22, and -23 and STAT3 on signal transduction pathways and their inhibition in autoimmune arthritis

**DOI:** 10.1007/s12026-021-09173-9

**Published:** 2021-01-29

**Authors:** Izabela Woś, Jacek Tabarkiewicz

**Affiliations:** 1 Laboratory for Translational Research in Medicine, Centre for Innovative Research in Medical and Natural Sciences, College for Medical Sciences of University of Rzeszow, ul. Warzywna 1a, 35-310 Rzeszow, Poland; 2 Department of Human Immunology, Institute of Medical Sciences, College for Medical Sciences of University of Rzeszow, ul. Warzywna 1a, 35-310 Rzeszow, Poland

**Keywords:** Autoimmune arthritis, Interleukin, STAT3, Anti-inflamatory drugs

## Abstract

Rheumatic diseases are complex autoimmune diseases which include among others rheumatoid arthritis (RA), juvenile idiopathic arthritis (JIA), and psoriatic arthritis (PsA). These diseases are characterized by prolonged and increased secretion of inflammatory factors, eventually leading to inflammation. This is often accompanied by persistent pain and stiffness in the joint and finally bone destruction and osteoporosis. These diseases can occur at any age, regardless of gender or origin. Autoimmune arthritis is admittedly associated with long-term treatment, and discontinuation of medication is associated with unavoidable relapse. Therefore, it is important to detect the disease at an early stage and apply appropriate preventative measures. During inflammation, pro-inflammatory factors such as interleukins (IL)-6, -17, -21, -22, and -23 are secreted, while anti-inflammatory factors including IL-10 are downregulated. Research conducted over the past several years has focused on inhibiting inflammatory pathways and activating anti-inflammatory factors to improve the quality of life of people with rheumatic diseases. The aim of this paper is to review current knowledge on stimulatory and inhibitory pathways involving the signal transducer and activator of transcription 3 (STAT3). STAT3 has been shown to be one of the crucial factors involved in inflammation and is directly linked with other pro-inflammatory factors and thus is a target of current research on rheumatoid diseases.

## Introduction

Autoimmune diseases are a group of multifactorial disorders with complex and unclear etiology. Autoimmune rheumatic diseases are characterized by similar pathophysiological mechanisms and risk of systemic complications such as cardiovascular diseases, osteoporosis, and early death [1, 2]. Clinical syndromes are initiated by an abnormal immune response that incorrectly reads self antigens as foreign, attacking the body and leading to inflammation.

The immune system produces autoantibodies directed against self antigens. This process, called autoimmunity, is the basis for autoimmune diseases [3]. To date, two ways of autoimmunity development are known. Firstly, physiological autoimmunization may be characterized by a lack of clinical symptoms, i.e., the production of autoantibodies, and at this point the body’s homeostasis is maintained by elimination of degraded self and non-self antigens. The second process is associated with the occurrence of pathological autoimmune reactions that causes tissue damage. The occurrence of genetic predisposition and/or environmental factors (e.g., smoking, UV light, heavy metals) with increased activation of autoreactive T and B lymphocytes leads to tissue damage and loss of their functions [4, 3].

The occurrence of inflammation results from a complex relationship between genetics, hormonal, epigenetics, and environmental factors [1]. The association between the severity of rheumatoid arthritis and the expression of the major histocompatibility complex (MHC), known in humans as human leukocyte antigens (HLA), has been described. The HLA alleles that predispose to the disease, in addition to HLA alleles protecting against autoimmune diseases, are present [5].

Hypothalamic-pituitary-adrenal (HPA) immune axis dysfunction could participate in autoimmune arthritis pathogenesis. During increased inflammation, there is not enough cortisol produced by the adrenal glands axis [6]. The pathomechanism of environmental factors, smoking [7], obesity [8], malnutrition with vitamin D deficiency, and environmental toxins such as heavy metals, infections, and drugs, on the occurrence of RA remains unclear [9, 8, 10]. Epigenetic disorders, related to changes in gene expression, may result from the environmental impact on humans. The epigenome is sensitive to environmental factors. Lowering of DNA methylation in T cells and peripheral blood mononuclear cells (PBMCs) or modification of histone proteins, described in detail by Araki and Mimura [11], may influence modifications of genes responsible for induction and maintenance of inflammatory processes in joints [12–14].

Arthritis is characterized by hyperplasia of the synovium, resulting in increased secretory activity. Synovial fluid, produced in large quantities, is characterized by an abnormal chemical composition, leading to dysfunction. T and B cells, macrophages, and synovial fibroblasts infiltrate the synovium. T helper type 17 (Th17) cells play a pivotal role in RA and under the influence of IL-1, IL-6, IL-23, and transforming growth factor β (TGFβ) differentiate from naïve CD4+ T cells. Th17 cells are characterized by surface markers, i.e., CD4, CD161, IL-6, and IL-23 receptors and chemokine receptors CCR4 and CCR6 [15, 16]. Th17 cells express the retinoic acid receptor–related orphan receptor gamma t (RORγt), which is involved in the differentiation of naïve CD4+ T cells into Th17. Lack of this factor inhibits IL-17 production because it causes a defect in Th17 cell development [17]. Th17 cells release IL-8, IL-17, IL-21, IL-23, chemokine CCL20, and granulocyte-macrophage colony-stimulating factor (GM-CSF), with simultaneous inhibition of production of anti-inflammatory factors, i.e., IL-10. Additionally, activated B cells release autoantibodies, rheumatoid factor (RF), which is directed against the Fc part of human IgG [1, 16], and anti-citrullinated protein antibody (ACPA). Protein citrullination occurs through enzymatic catalysis of peptidylarginine residues into peptidylcitrulline involving peptidylarginine deiminase (PAD). This is a characteristic of tissue with inflammation; however, not all patients have been shown to produce ACPA. ACPA+ patients are characterized by more aggressive disease progression; hence, ACPA is considered to be an indicator of RA development [1, 18]. Arthritis is characterized by angiogenesis and osteoclastogenesis, as well as systemic disorders including cardiovascular and pulmonary dysfunctions [1, 10].

Class II cytokines, including the interferons (α, β, γ, ε, κ, λ, ω) and IL-10 family, appear to be effective therapeutic measures in restoring immune homeostasis and preventing disease relapses common in RA patients [19, 20]. IL-10 belongs to the class II cytokines including its homologs: the IL-20 subfamily (IL-19, IL-20, IL-22, IL-24, IL-26) and the IL-28 subfamily (IL-28A, IL-28B, IL-29). These cytokines are produced by innate and adaptive immune cells and are involved in inflammation, infection, autoimmunity, and tissue homeostasis. The Janus tyrosine kinase (JAK)/STAT pathway activated by the respective receptors plays a key role in the signaling of type II cytokines. Additionally, receptors such as IL-22R or the type I interferon receptor (IFNR) activate the mitogen-activated protein kinase (MAPK) pathways [21].

We will discuss the role of cytokines and chemokines produced during arthritis and the pathway in which they participate, as well as their impact on tissues and the ability to inhibit these pro-inflammatory agents by using inhibitors acting on inflammatory mediators. Due to the variety of possible inflammatory factors and the limited numbers of comprehensive papers, we focused on the key factors associated with pathogenic differentiation of Th17 and Th22 cells and IL-17 and IL-22 production in course of autoimmune arthritis selecting IL-6, -21, and -23 and STAT3 for review. Also, the possibility of targeting these mediators for therapetic purposes was a key factor for their selection. The prospect of further research should focus on the balance between pro- and anti-inflammatory cytokines, and the possibility of increasing the secretion of anti-inflammatory cytokines and inhibiting pro-inflammatory factors.

## Interleukin-6

IL-6 is produced by macrophages, dendritic cells, neutrophils, mast cells, B lymphocytes, and by some CD4+ effector cells. In response to environmental and intracellular signals, IL-6 is also produced by non-immune cells such as fibroblasts, and endothelial and epithelial cells [22]. IL-6 consists of a four α-helical bundle and triggers the JAK/STAT and the Ras/MAPK pathways by binding to a specific IL-6 receptor (IL-6R) consisting of IL-6-specific α receptor (IL-6Rα) and to glycoprotein 130 (gp130) signal transducer. IL-6 binds to a transmembrane or soluble form of IL-6Rα that can further interact with gp130 to induce signal transduction and gene expression. Homodimerization of gp130 leads to phosphorylation of the JAKs family (JAK1, JAK2, TYK2), as well as recruitment and activation of STAT1 and STAT3 [23–25], by which proteins are secreted, including C-reactive protein (CRP) and haptoglobins, which trigger inflammatory reactions [26]. IL-6 is an essential cytokine that transports acute phase responses and immune responses to prepare for host defense. However, current studies show that long-term and excessive IL-6 production leads to severe inflammation [27]. IL-6 and tumor necrosis factor-α (TNF-α) are particularly involved in joint inflammation because they lead to activation of synoviocytes that directly influence the extracellular matrix (ECM) and secrete matrix metalloproteinases (MMPs) into the synovial fluid leading to degradation of cartilage, bone, and, consequently, joints [28]. TNF-α is one of the crucial targets in autoimmune arthritis therapy. It is a cytokine that has been studied for many years against which a significant number of agents are known, such as the monoclonal antibodies adalimumab, infliximab, and the fusion protein etanercept (ETA) [29]. Several reviews have considered anti-TNF-α therapy and our focus will be on other therapeutic targets and pro-inflammatory pathways that are not clearly understood.

The role of IL-6 in the pathogenesis of RA is confirmed, but it remains unclear how it specifically promotes autoimmunity and leads to tissue damage. IL-6 and TGFβ upregulate RORγt expression, which is required to promote production of Th17 cells and IL-17 expression. TGFβ, IL-1β, IL-6, IL-21, and IL-23 are key factors for differentiation of CD4+ T cells to Th17. In addition to IL-6, IL-21 and IL-23 can induce STAT3, and both of these compounds take part in arthritis [30]. IL-6 is often used as a marker of rheumatoid arthritis [22].

Increased levels of IL-6 correlating with disease severity have been marked in the serum as well as in the synovial tissue where the main source of this interleukin is fibroblast-like synoviocytes in RA (RA-FLS) [22]. Research confirms that IL-6 has a pivotal role for STAT3 activation in CD4+ T cells, especially for pathogenesis of ACPA-negative arthritis [31]. Clinical studies have shown that tocilizumab, a humanized monoclonal antibody against the IL-6 receptor, is efficient in treating RA [32]. Studies have not shown any changes in the incidence of circulating monocytes, T or B cells. Also, tocilizumab did not affect the production of interferon-γ (IFNγ) and IL-17 by memory/activated CD4+ cells with a non-significant reduction in IL-2 production. However, IL-6 blocking has been observed to reduce the production of IL-21 by activated/memory CD4+ T cells, as well as decreased levels of IL-21 mRNA in freshly isolated cells. Furthermore, blockage of IL-6 was also associated with decreased production of IgG, mainly IgG4 [22]. Tocilizumab is also safe and efficient for patients with systemic JIA, but incidence of serious hypersensitivity reactions were observed [33]. Clinical studies have shown that IL-6R blockade with tocilizumab (8 mg per kg bodyweight every 4 weeks) or sarilumab (human anti-IL-6R monoclonal antibody (150–200 mg every 2 weeks)) is more effective than TNF-α blockade by the anti-TNF-α blocking antibody (adalimumab) in patients with RA. In the studies comparing the effectiveness of these drugs, patients received 40 mg of adalimumab every 2 weeks [34–36].

Differences in the IL-6 signal inhibition strength in various patients demonstrated that the signaling pathways may be different in each patient, and use of intervals in tocilizumab administration was of noted importance IL-6 transmits signals by phosphorylation of STAT3, but also STAT1 and STAT5 (phospho-STAT). Evaluation of the percentage of pSTAT3-positive CD4+ T cells stimulated with IL-6 can function as a new method for demonstrating the IL-6/STAT3 signal inhibition strength. However, as the authors suggest, this method has some limitations and its clinical usefulness should be confirmed [37, 38]. Currently, extensive studies have demonstrated the efficacy of tocilizumab, both as a monotherapy and in combination with conventional synthetic disease-modifying anti-rheumatic drugs (csDMARDs) in adults with early and prolonged RA. Tocilizumab, intravenous (IV) or subcutaneous (SC) infusion, was the first drug approved by the Food and Drug Administration (FDA) respectively in 2010 and 2012, which resulted in rapid and sustained improvement in patients with RA. Tocilizumab was approved for use in children with polyarticular JIA as SC in 2013 [26, 39, 40].

Sirukumab, a human anti-IL-6 monoclonal antibody in patients with RA, has also been studied. Although sirukumab was safe and well-tolerated by patients with active RA, refractory or intolerant DMARDs, and improved symptoms of the disease, the FDA did not approve this drug for the treatment of RA in 2017. Some effectiveness in the therapy of rheumatoid arthritis has been demonstrated, but future research is required due to multiple side effects [41–44].

**Fig. 1:**
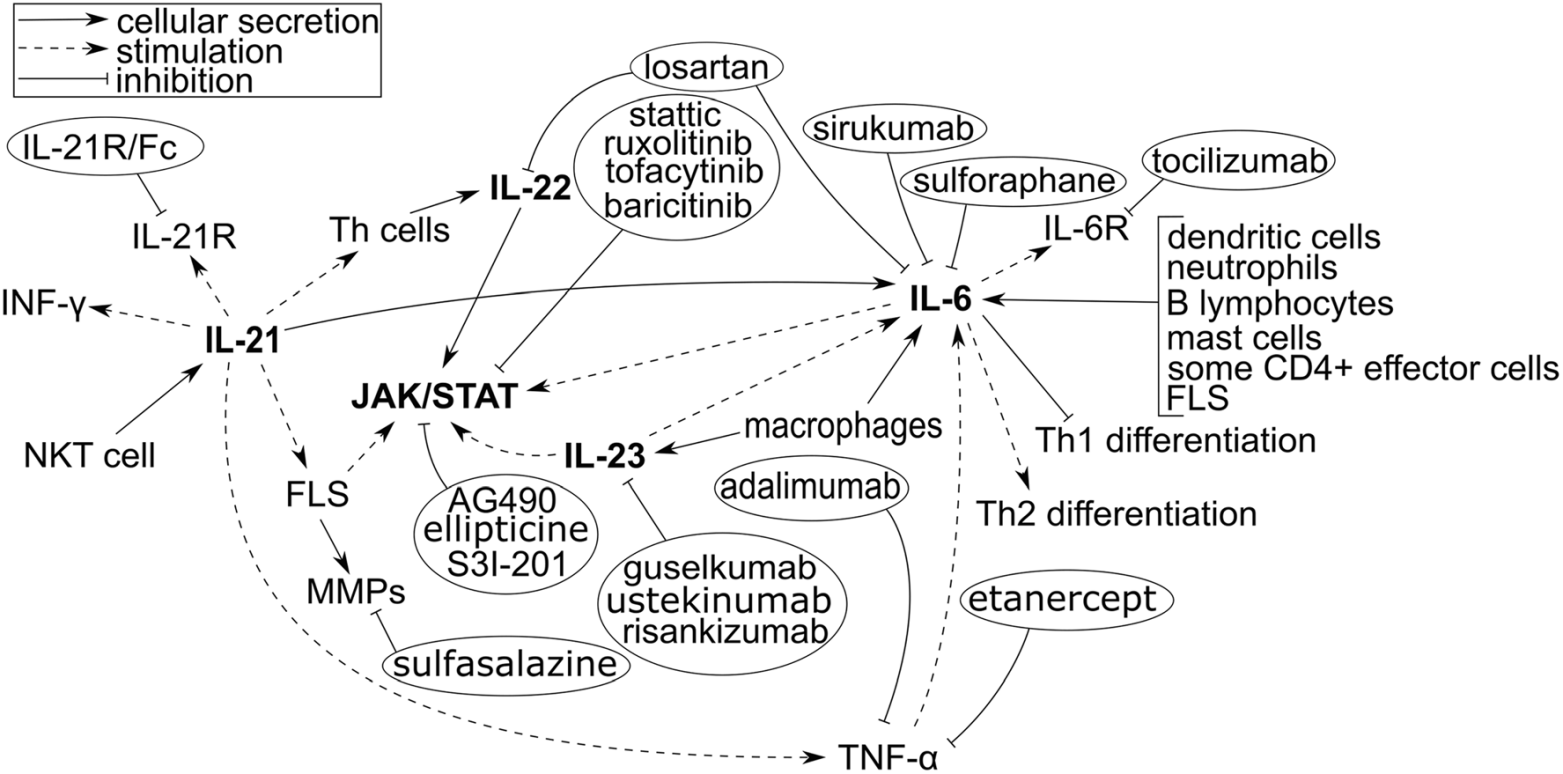
The major signal transduction pathway via IL-6, IL-21, IL-22, IL-23, and therapeutic monoclonal antibodies that inhibit the function of interleukins; IL-6R: IL-6 receptor; IL-: interleukin-; Th cells: T helper cells; INF-γ: interferon-γ; MMPs: matrix metalloproteinases; TNF-α: tumor necrosis factor-α; FLS: fibroblast-like synoviocytes; JAK/STAT: Janus kinase/signal transducers and activators of transcription

Figure 1 shows the most important IL-6 signaling pathways discussed in this paragraph along with anti-inflammatory drugs, but IL-21, IL-22, and IL-23 (discussed in the following paragraphs) are also included due to their cross-linking.

## Interleukin-17

IL-17 is produced by Th17 subset of CD4+ and CD8+ T cells [45, 46]. IL-17 is produced also by Tγδ cells, natural killer (NK) cells, natural killer T (NKT) cells, mast cells, and neutrophils [47, 48]. IL-17 includes six isoforms (A-F) of which IL-17A is best-characterized with confirmed pro-inflammatory potential [46]. The receptor family of the IL-17 consists of five members (IL-17RA, IL-17RB, IL-17RC, IL-17RD, IL-17RE, IL-17RF) of which the best known are IL-17A, IL-17F, and IL-17A/F signaling pathways. IL-17R consists of homodimers or heterodimers of IL-17RA and IL-17RC. Receptors consist of a conserved cytoplasmic SEF/IL-17R (SEFIR) domain, an outer membrane fibronectin III-like domain, and a distal activation domain (CBAD). After stimulation by IL-17, Act1 protein is recruited to receptors by two SEFIR domains leading to activation signaling cascades of the nuclear factor κ-light-chain-enhancer of activated B cells (NF-κB), MAPK, and CCAAT/enhancer binding protein (C/EBP) pathways and triggering the transcription of IL-17 target gene [47, 49, 50]. IL-17A and IL-17F are homodimeric glycoproteins with a cysteine-knot-like fold; however, they can also form heterodimers. Studies show that activated CD4+ T cells produce both homodimers and an IL-17A and IL-17F heterodimer that can activate fibroblasts and macrophages to produce IL-6 [51]. IL-17 is also an inductor of other pro-inflammatory cytokines GM-CSF, IL-1β, TNF-α, prostaglandin E_2_ (PGE2), and chemokines (CXCL1, CXCL2, CXCL5, CXCL8, CXCL10) [52, 15]. IL-17 increases the production of MMPs, degrading the matrix in synovial tissue and cartilage [53]. Kim et al. [54] suggested that inflammation associated with IL-17 can stimulate autophagy leading to mitochondrial dysfunction in RA-FLS. IL-17 stimulates activation of NF-κB involved in osteoclastogenesis and formation of pannus-abnormal growth of tissue development in joints [53], as well as neoangiogenesis of the synovium [55]. It is suggested that the role of IL-17 in inflammation may depend on the stage of the disease [56]. Raza et al. [57] showed that synovial fluid collected from patients at an early stage of the disease contains higher levels of cytokines, mainly IL-17, than in synovial fluid from patients with established disease. The study showed that IL-17R are expressed in RA-FLS and the IL-17/IL-17R/STAT3 pathway is critical for the proliferation and survival of synoviocytes. STAT3 has an important role in the differentiation of Th17 cells and upregulates IL-17 expression [58].

In humans, PBMCs, CCR6, and CCR4 are co-expressed on Th17 cells. CCR6 is involved in the recruitment of Th17 cells to sites of inflammation by the CCL20 ligand [59]. There are many contradictory studies concerning the involvement of CCL20 in arthritis. Some studies show that serum IL-17 and CCL20 levels decrease after anti-TNF-α therapy [60], while other studies have not confimed such a relationship [61]. Studies conducted by Clanchy and Williams [62] have shown a decrease in the expression of *CCL5* and *CCL3* by treatment with phosphodiesterase type 4 (PDE4) inhibitor (ibudilast) in activated RASFs. In addition, ibudilast also inhibited the expression and secretion of IL-12/23 p40, and Th17 cells responses *in vivo*.

Sulforaphane (SFN), an isothiocyanate originating from vegetables including broccoli with natural antioxidant with anti-inflammatory, anticancer, and antibacterial properties, was used to study arthritis inhibition [63]. SFN has been shown to induce apoptosis of synoviocytes by regulating the expression of Bcl-2/Bax, p53, and pAkt. SFN-inactivated pAkt induces changes in Bcl-2, p53, and Bax expression, promoting apoptosis. SFN can decrease RA-associated inflammation, by reducing the abnormal proliferation of synoviocytes. Non-apoptotic doses of SFN led to inhibition of T cell proliferation and IL-17 and TNF-α production by anti-CD3 antibody–stimulated RA CD4+ T cells. In addition, the production of IL-6, INF-γ, IL-17, and TNF-α by lymph node and spleen cells after SFN treatment has been shown to decrease. Thus, it can be concluded that SFN inhibits synovial hyperplasia, T cell proliferation, and the production of pro-inflammatory agents by RA T cells [64]. SFN in naïve synoviocytes induced cytoprotective transcription factor (Nrf2), and induced apoptosis in inflammatory TNF-α-stimulated synoviocytes. There was no inhibition of the activity or expression of MMP-3 or MMP-9 in TNF-α stimulated synoviocytes [65]. Choi et al. [66] showed that SFN inhibited the expression of MMP, COX-2, and PGE2 in synovial fibroblasts involved in synovitis. Studies have been carried out on murine models of complete Freund’s adjuvant (CFA)–induced arthritis and SFN administration and showed increased levels of IL-6 and activation of thioredoxin reductase. In addition, improvement in the treatment of arthritis has been shown by modulation of cell migration to the joints and their activation, as well as regulation of cytokine production [63]. At the same time, other groups demonstrated an inhibitory effect of SFN on the expression of IL-1, IL-6, and IL-8 in endothelial cells by the Rho/Rho-associated coiled-coil containing protein kinase (RhoA/ROCK) and NF-κB pathways [67, 68]. Inhibition of T cell activation by SFN decreased the expression of RORγt and inflammatory cytokines produced by Th17 cells (IL-17A, IL-17F, and IL-22) [69]. Other researchers have also shown that SFN inhibits expression of MMPs in mice with induced arthritis, and reduction of IL-17 and TNF-α levels in T cells from patients with rheumatoid arthritis [70]. These studies suggest that SFN may be a new therapeutic agent for autoimmune diseases in which Th17 cells are significant, e.g., rheumatoid arthritis [69], because isothiocyanates were detected in synovial fluid after broccoli intake having a beneficial effect on the joints [71].

The influence of obesity on the occurrence of arthritis was also examined. Obesity is associated with the presence of elevated levels of IL-1β, which affects induction and differentiation of Th17 pathogenic cells in patients with RA. In addition, these patients responded less well to TNF-α inhibitors. Therefore, it is suggested that anti-IL-1β, anti-IL-21, and anti-Th17 therapies are beneficial for obese people and, additionally, weight loss increases the response to anti-TNF therapies [8].

In research conducted by Kamel et al. [72], methotrexate (MTX) was used in rats with experimentally CFA-induced arthritis. MTX reduced joint damage and decreased levels of TNF-α, IL-6, IL-17, and MMP-3. The expression of the receptor activator of nuclear factor κΒ ligand (RANKL), STAT3, vascular endothelial growth factor (VEGF), and NF-κB genes was also decreased. Therefore, it seems appropriate to acknowledge the IL-6/STAT3/IL-17/NF-κB signaling cascade as a modulator of arthritis and potential therapeutic target. Sulfasalazine, a synthetic DMARD characterized by high efficacy and low toxicity profile is approved for the treatment of arthritis by inhibiting NF-κB [73].

Research has also confirmed the effect of the JAK2/STAT3 pathway on regulating the activity and differentiation of osteoblasts from patients with ankylosing spondylitis (AS). IL-17A correlated with osteoblast differentiation and blocking of this cytokine inhibited JAK2/STAT3 phosphorylation [74]. The fact that the STAT3 pathway is involved in inflammation has also been demonstrated [75]. There are various therapies directed against IL-17 (secukinumab, ixekizumab, bimekizumab), and against the IL-17 receptor (brodalumab).

Secukinumab is a fully human anti-IL-17A monoclonal antibody preventing receptor binding and inhibiting the induction of an inflammatory response. The latest research confirms and extends previous results that show secukinumab is safe, clinically effective, and that low rates of radiological progression were maintained for patients with PsA [76]. Treatment was associated with low immunogenicity in patients with PsA [77]. Secukinumab has also shown efficacy and safety in treating RA in patients who have had an inadequate response to TNF inhibitors [78].

In 2020, studies on the effects of treatment with ixekizumab from clinical trials on patients with PsA have been published. Ixekizumab is a recombinant humanized monoclonal IgG subclass 4-κ (IgG4-κ) antibody that selectively binds and neutralizes IL-17. Studies have shown a positive effect on the inhibition of IL-17A in PsA and the results were consistent with the known safety profile of ixekizumab [79, 80].

The study also used bimekizumab, which is a selective monoclonal antibody that inhibits the activity of IL-17A and IL-17F. Patients with PsA showed a positive response to treatment with this antibody, and brought new therapeutic benefits in the treatment of this disease [81]. In 2017, it was shown that bimekizumab is safe, well tolerated, and has positive clinical properties in the inhibition of IL-17A and IL-17F in patients with mild psoriasis. These studies support further development of this drug as a therapeutic for diseases dependent on these cytokines [82]. This study was confirmed by Glatt et al. [83] by demonstrating that bimekizumab is safe as an add-on therapy for RA patients with inadequate certolizumab pegol response.

Brodalumab is an antibody directed against the IL-17A receptor. Studies have not shown clinical efficacy of brodalumab in the treatment of RA in people who have had an inadequate response to MTX; however, improvements in clinical outcomes have been observed in patients with PsA [84].

The literature also presents research on the impact of chemical compounds on RA. The research focused on an assessment of the effect of sodium chloride (NaCl) on differentiation of Th17 cells. Mice with collagen-induced arthritis (CIA) underwent diets with normal (group I) and with large (group II) salt contents. Increased IL-17 expression was demonstrated in CIA mice and increased Th17 differentiation in a dose-dependent manner. This study showed influence of a salt-rich diet as one of the possible causes of arthritis or its severity [85]. Similar studies were conducted in 2013 by Kleinewietfeld et al. [86].

**Fig. 2:**
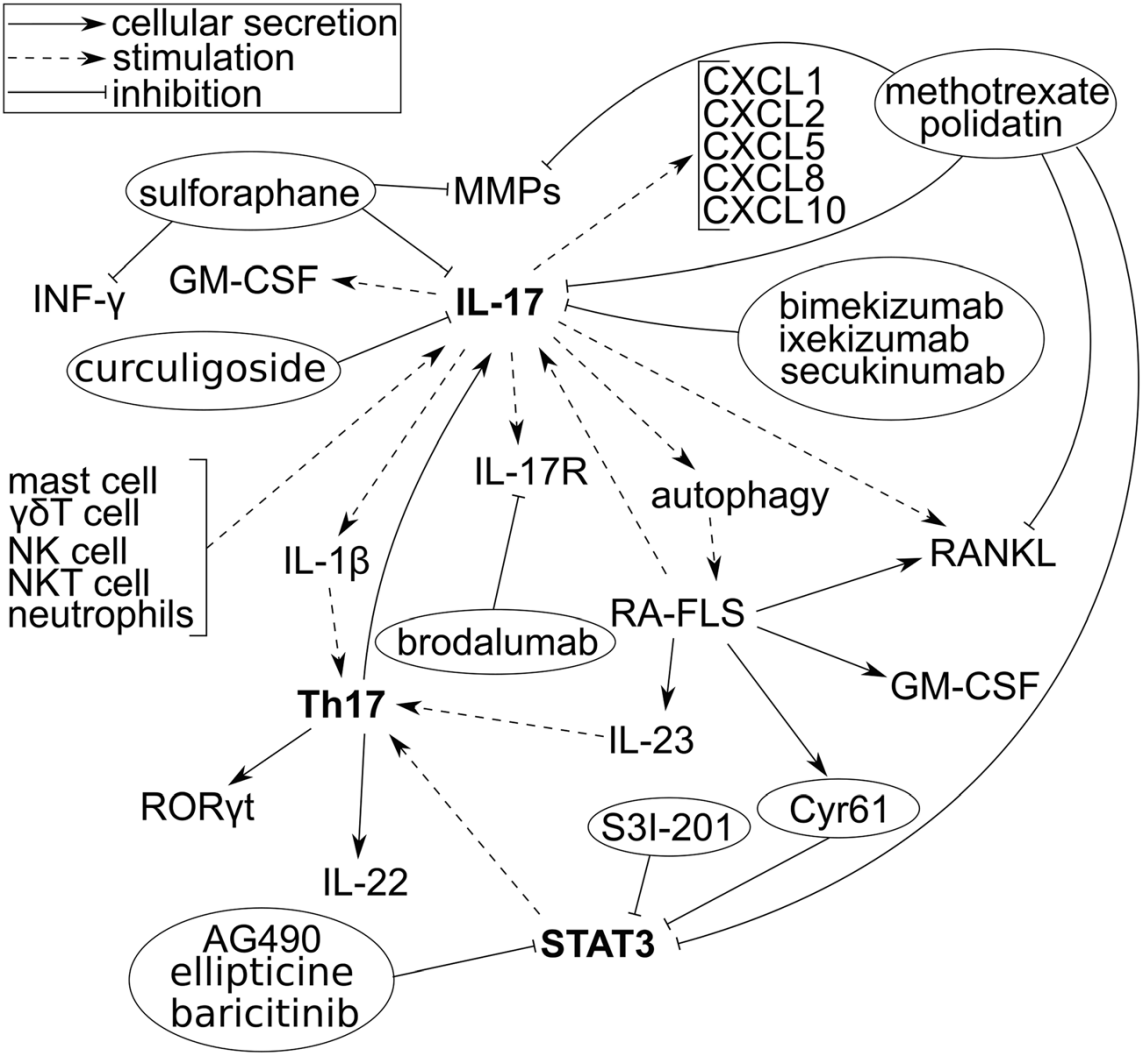
The major signal transduction pathway via IL-17 and therapeutic monoclonal antibodies that inhibit IL-17; IL-: interleukin-; IL-17/21R: IL-17/21 receptor; Th17 cells: T helper type 17 cells; INF-γ: interferon-γ; MMPs: matrix metalloproteinases; FLS: fibroblast-like synoviocytes; STAT3: signal transducers and activators of transcription 3; GM-CSF: granulocyte-macrophage colony-stimulating factor; RORγt: retinoic acid receptor-related orphan receptor gamma t; RANKL: receptor activator of nuclear factor κΒ ligand; CXCL-: chemokine (C-X-C motif) ligand-; Cyr61: cysteine-rich angiogenic inducer 61; NK cells: natural killer cells; NKT cells: natural killer T cells

Figure 2 shows the major signal transduction pathway for IL-17 discussed in this paper along with anti-inflammatory drugs.

## Interleukin-21

IL-21 is a cytokine produced by activated T helper cells (Th1, Th2, Th17), T follicular helper (Tfh) cells, and NKT cells. It belongs to the family of type I cytokines containing four α-helical structures [28, 87]. IL-21 is overproduced in intestinal inflammation, psoriasis, type I diabetes, systemic lupus erythematosus (SLE), and RA. IL-21 is a cytokine that plays a very important role in the control of immune-mediated diseases. This cytokine is characterized by its dual role, having pro-inflammatory and anti-inflammatory properties. Studies showed that IL-21 may protect mice against colitis [87, 88]. IL-21 is involved in the normal differentiation of B cells into plasma cells and production of immunoglobulins, T cell differentiation by co-stimulation with anti-CD3, and development of Th17 cells and Tfh cells [89]. IL-21 may serve as a biomarker for the detection of disease activity [90]. Clinical studies on the effects of IL-21 in solid tumors have produced beneficial results in treatment; yet, on the other hand, IL-21 promotes autoimmune diseases. IL-21 is a pleiotropic cytokine, regulating both innate and adaptive (humoral and cellular) immune responses, affecting a wide range of lymphoid, myeloid, and epithelial cells [91]. IL-21 binds to the IL-21R complex consisting of IL-21R and the γ-chain, common to receptors specific for cytokines of the IL-2 family: IL-2, IL-4, IL-7, IL-9, IL-15. Stimulation activates JAK1 and JAK3 which phosphorylates STAT1, STAT3, and STAT5. IL-21/IL-21R signaling can lower inflammation and alleviate the symptoms of disease [28, 87]. Studies performed by Li et al. [92] showed that IL-21R was expressed in CD4+ or CD8+ T cells, B, and NK cells. IL-21R expression is elevated in the synovium, and also in RA-PBMCs. IL-21 may increase the activation of T cells locally and proliferate secretion of pro-inflammatory cytokines and may be closely related to the occurrence of RA. IL-21 has also been shown to be an activator of secretion of TNF-α, INF-γ, and other cytokines by T cells in RA patients. IL-21 neutralization by the IL-21/Fc receptor (IL-21R/Fc) fusion protein can inhibit the production of proinflammatory factors. Administration of IL-21 prior to induction of experimental autoimmune encephalomyelitis (EAE) led to enhanced NK cell function and IFN-γ secretion. Lowering the number of NK cells leads to the abolition of IL-21 action [93].

In inflammation, RA-FLS play an important role. They induce activation and accumulation of inflammatory cells and synthesize growth factors that stimulate angiogenesis. RA-FLS can also migrate from the patient to healthy synovium. Xing et al. [87] showed that the cytokine IL-21 influences the migration, invasion, and production of MMPs in RA-FLS (MMP-2, MMP-3, MMP-9, MMP-13). Additionally, they confirmed that IL-21 induced activation of JAK/STAT, and phosphatidylinositol 3-kinase (PI3K)/Akt, MAPK/extracellular signal regulated protein kinases 1 and 2 (ERK1/2) pathways. Inhibition of each kinase pathways appropriately by STA-21, LY294002, and PD98059 attenuated IL-21-induced migration of RA-FLS and secretion of MMP-3 and MMP-9; however, inhibition of the STAT3 by AG490 had no influence on IL-21-induced RA-FLS migration. Another study reported that inhibition of the JAK/STAT3 pathway by AG490 suppresses hyperactivation of osteoblasts induced by serum from patients with AS [74]. Activation of the kinase pathways were detectable after stimulation induced directly by IL-21. Blockage of kinase pathways inhibited IL-21-induced RA-FLS proliferation and secretion of IL-6 and TNF-α by RA-FLS. It can therefore be assumed that IL-21 promotes the secretion of IL-6 and TNF-α by activating respectively the STAT3, ERK1/2, and Akt in RA-FLS, and inhibition of these pathways could be a future direction of arthritis research [28]. MMPs are molecules involved in the degradation of the extracellular matrix (ECM) and basal membranes, causing the migration of various cells. Researchers have shown that IL-21 stimulates MMPs production by RA-FLS. Blocking of the signaling pathway through the IL-21R/Fc inhibited secretion of MMP-3 and MMP-9 in RA-FLS induced by this cytokine. Further studies on blocking IL-21 signaling by IL-21R/Fc reduced cytokine production and arthritis in animal models of CIA [94]. IL-21 did not affect the expression of tissue inhibitor metalloproteinases 1 and 2 (TIMP-1 and TIMP-2) in RA-FLS stimulated with IL-21. IL-21 regulated the expression of intercellular adhesion molecules 1 (ICAM-1) and cadherin-11 [87]. Studies have shown a positive correlation between autoantibodies (AAbs) against IL-21 and disease activity score 28 joints (DAS28). IL-21 and anti-IL-21 AAbs have been detected in RA but no correlation has been demonstrated; therefore, future research is needed to explain the influence of IL-21 and anti-IL-21 AAbs on the pathogenesis and progression of RA. Research carried out on IL-21 is not always consistent, which may result from the variability of the course of the disease, clinical characteristics, ethnicity, or sensitivity of enzyme-linked immunosorbent assay (ELISA) kits [95]. In a study by Sglunda et al. [90], IL-21 and IL-23 levels in plasma after 12 weeks of treatment correlated with DAS28 and erythrocyte sedimentation rate (ESR). Research showed that patients after 12 weeks of treatment DMARDs and/or glucocorticoids showed a reduced level of IL-21. Levels in treated individuals were even lower than in healthy people, which may be due to reduced Th17 cell proliferation, which is associated with decreased production of IL-21 by cells. Studies conducted by Sakuraba et al. [96] have shown that IL-21 signaling in B cells (expression of the IL-21 receptor on B cells) is crucial for the development of CIA in an animal model of IL-21 receptor knockout *Il21r* KO mice. It was also shown that in RAW264.7 cells that do not express the RANKL, IL-21 promoted osteoclastogenesis regardless of the prevalence of RANKL (as suggested by previous studies). The osteoclastogenic potential is dependent on the PI3K/Akt signaling pathway, because the use of the PI3K/Akt pathway inhibitor (LY294002) significantly inhibited IL-21 induced osteoclastogenesis [97].

## Interleukin-22

IL-22 is an α-helical cytokine belonging to the IL-10 cytokine superfamily. The human *IL-22* gene is found on the 12q15 chromosome in addition to the *IFN-γ* and *IL-26* genes [98], and is produced by Th17 [56] and Th22 cells. The production of IL-22 is promoted by IL-17, IL-23, IL-1β, aryl-hydrocarbon receptors (AhR), and Notch signaling [99]. The IL-22R is a complex of IL-22R1 and IL-10R2 containing an intracellular, transmembrane, and extracellular signaling region. The cytokine binds to IL-22R1 leading to the formation of a complex. The IL-22/IL-22R1 complex changes conformation and allows association of IL-10R2, initiating the activation of tyrosine kinases 2 (TYK2) and JAK1, followed by phosphorylation of STAT3 on the tyrosine and serine residues, STAT1 and STAT5. It is also an activator of the MAPK pathways (ERK1/2, MEK1/2, c-Jun N-terminal kinase (JNK), and p38 kinase), which ultimately leads to antibacterial and inflammatory processes as well as tissue repair, depending on the environment in the organism in which the cytokine is expressed [100]. There is data on the duality of IL-22 activity in the literature which show the pro-inflammatory role of IL-22. On the other hand, there is also data on the protective role of IL-22 in controlling lung epithelial damage [101] or intestinal inflammation.

IL-22 levels are elevated in patients with rheumatoid arthritis and there is a relationship between its level and radiographic progression and disease activity [102, 103]. Researchers have shown that sulforaphane has an effect on increasing the levels of ROS in whole blood lymphocytes in RA patients. At the same time, reduced production of pro-inflammatory cytokines, i.e., IL-17A, IL-17F, and IL-22, has been demonstrated [69]. Studies conducted by Liu et al. [104] have shown that norepinephrine (NE), a neurotransmitter released from sympathetic nerves, inhibits the differentiation and function of Th17 cells by activating the β2-adrenergic receptor (β2-AR) on CD4+ T lymphocytes. The studies were conducted on CIA mice. This suggests that NE may have anti-inflammatory effects in CIA. A study was also carried out on rats suffering from pristane induced arthritis (PIA). Increased cytokines produced by Th17 (IL-17A, IL-21, IL-22), mainly IL-22 in the ratio of Th1 cytokines (TNF-α, INF-γ) and Th2 (IL-4, IL-10, TGFβ), have been shown in organs of immune rats (inguinal lymph nodes, spleen). Expression of IL-22 in synovium and serum correlated with the severity of PIA. The concentration of IL-21 was higher in PIA rats but was not significant compared to IL-22. In this study, IL-21 only supported Th17 differentiation and enhanced their response [99]. The same group showed that in PIA rats, the level of IL-22 expression was different in different phases of PIA. IL-22 levels increased in the spleen during the initial and chronic phase and in the synovium in the chronic phase. In contrast, no elevated levels of IL-22 were found in the acute phase of inflammation. In the acute phase, an increase in IL-17F and IFN-γ expression was observed in the synovial membrane of PIA rats [105]. Zhong et al. [106] reports that elevated IL-22+ T cells and IL-22 can promote RA development. Targeting Th22 and Th17 positively influences RA therapy. Patients were divided into two groups after basic treatments using conventional DMARDs, MTX, and leflunomide. The decreased plasma level of IL-22 correlated with a decreased level of Th22 and positively correlated with the reduction of DAS after treatment. The involvement of these cells in the pathogenesis of RA was previously demonstrated [107]. It has also been shown that treatment with MTX or ETA improves sleep efficiency because RA can cause sleep problems with a noted involvement of the HPA axis [108].

Studies were carried out on FLS from RA patients treated with sodium nitroprusside, inducing apoptosis in the presence or absence of IL-22. IL-22 has been shown to increase the viability of RA-FLS and prevent apoptosis. STAT3 inhibitors reversed this process. Studies have shown that IL-22 protects against sodium nitroprusside-induced apoptosis in RA-FLS by activating STAT3 and the *Bcl-2* gene [109]. The effect of STAT3 signaling on RA-FLS proliferation with the help of IL-22 has also been shown on research conducted by Zhu et al. [110]. It can therefore be assumed that the IL-22/STAT3 pathway may influence the pathogenesis of RA, with particular emphasis on the impact on RA-FLS survival, which may be one of the RA therapeutic pathways. Inhibition of IL-22 production is also important due to the effect on the management of osteoporosis. Studies have shown that Th22 cells express CCR10; therefore, their migration activity is directed towards the presence of CCL28 ligand, which occurs in the inflammatory synovium membrane. In this situation, Th22 cells infiltrate the synovium to produce IL-22, which additionally enhances inflammation. Th22 affects the differentiation of osteoclasts. An increased amount of osteoclasts can lead to osteoporosis, and their severity depends on the cytokines secreted by T lymphocytes [111]. The results of research by Kim et al. [112] show the upregulation of RANKL by IL-22 in RA synovial fibroblasts leads to osteoclastogenesis. These processes carry out via including JAK/STAT signaling pathway. Wen et al. [113] showed that 1,25-dihydroxy vitamin D_3_ may induce the inhibitory effect on osteoclastogenesis and decreasing RANKL expression in RA-FLS. In this process, as mentioned before, IL-22 induces the activation of the JAK/STAT signaling.

Studies conducted by Cardoso et al. [114] on PBMCs from RA patients have shown that losartan, an antihypertensive drug, is an effective inhibitor of PBMC secretion by pro-inflammatory cytokines. Losartan at a concentration of 100 μM decreased the levels of IL-6, IL-17F, IL-22, and INF-γ, secreted by PBMC cultures. These studies have shown that hypertensive drugs can have two effects: as drugs for cardiovascular disease and anti-inflammatory in rheumatoid arthritis; therefore, losartan may be the best choice for people suffering from hypertension that affects 70% of patients with RA. Enalapril and valsartan were also examined, but did not show immunomodulatory effect. On the other hand, another study showed positive effects of enalapril on arterials stiffness in RA patients [115].

## Interleukin-23

IL-23 is a member of the IL-12 cytokine family (including IL-27, IL-35, IL-39). IL-23 is a heterodimer consisting of a p40 subunit covalently linked to the p19 subunit. IL-23 is produced by dendritic cells (DCs) and activated macrophages thus by activated antigen-presenting cells. IL-23 plays important role for Th17 cell development and maintenance [116] but also affects the pathogenicity of Th17 cells through the interaction with IL-17 and TNF-α [117]. IL-23 binds to its receptor consisting of IL-23R and IL-12Rβ1 subunits. IL-23/IL-23R complex recruited IL-12Rβ1 leading to phosphorylation of JAK2 and TYK2 followed by STAT3 and STAT5 [116]. Studies have shown that DLN (cultured draining LN (DLN) cells) cultured with the addition of IL-23 produced elevated amounts of IL-17 by Th17 and led to inflammation [118]. IL-23, similarly to IL-22, can have a dual role, both pro-inflammatory and anti-inflammatory. Research suggests that IL-23 can act as a biomarker in diagnosing RA due to elevated cytokine levels during inflammation [119]. IL-23 can induce differentiation of Th17 cells, increasing IL-17 levels and leading to osteoclastogenesis via the RORγt/STAT3 signaling pathway [120].

Ganesan and Rasool [121] showed that RA-FLS produce factors affecting the deterioration of the disease, i.e., cysteine-rich angiogenic inducer 61 (Cyr61), IL-23, GM-CSF, and RANKL. IL-23 expression was increased in a rat model of arthritic fibroblast-like synoviocytes (AA-FLS) at the protein and mRNA levels after IL-17 treatment. Blockade of STAT3 using S3I-201 reduced the production of IL-23 in AA-FLS; therefore, it can be stated that IL-23 production is dependent on STAT3. In addition, the JAK/STAT pathway contribution was confirmed in studies of Raychaudhuri et al. [122]. They observed that the production and regulation of IL-17 by Th17 cells is associated with the JAK/STAT pathway induced by IL-23. Tofacitinib may inhibit JAK, and thus also the pathway itself, contributing to inhibition of inflammation.

Studies conducted by Pfeifle et al. [123] have suggested that the transition of autoimmunity to clinical disease occurs through activation of the IL-23/Th17 axis. IL-23 did not affect inflammation but the inflammatory activity of autoantibodies that were newly produced; therefore, targeting this axis may be effective while maintaining clinical remission. Studies on IL-23 levels in people with early RA have been conducted earlier by Andersen et al. [124]. It has been shown that IL-23 levels decrease after receiving adalimumab for patients, which is significant in the preclinical development of RA. Future research may also focus on targeting the IL-23 signaling pathway, due to its involvement in disease relapse through a role in reactivation of memory T cells [125]

Ustekinumab, a human IgG1κ monoclonal antibody that binds to the p40 subunit common to IL-12 and IL-23, was approved for the treatment of PsA in 2013 [126]. Studies have shown that agents that reduce p19 binding to IL-23R may be used to inhibit IL-23 in inflammation, thus inhibiting the p19/IL-23-associated inflammation axis [127]. A phase 3 trial showed that the human monoclonal antibody guselkumab against p19 IL-23 was safe and effective in the treatment of patients with PsA [128]. The treatment of PsA patients also included risankizumab, an anti-IL-23A humanized IgG1 monoclonal antibody that binds to the p19 subunit [129].

IL-23 plays an important role in the pathophysiology of RA. Research also focuses on cytokine receptors and suggests that single-nucleotide polymorphisms (SNPs) of the *IL-23R* gene play an important role in RA. The *IL-23R* gene is located on human chromosome 1 (1p31). IL-23 activates the JAK/STAT signaling pathway by binding to the receptor and affects the transcription of the IL-17, IL-21, and IL-22 [130]. One of the better known SNP in the *IL-23R* gene is R381Q polymorphism, located between the putative JAK2 binding site and the trans-membrane domain in the cytoplasmic region of IL-23R protein. R381Q appears to play a protective role against the development of RA and has been shown to modulate IL-17A expression [131]. Gene function studies may be helpful in designing more effective immunological drug therapy for patients with immune diseases.

## JAK/STAT pathway

Activation of the JAK/STAT pathway plays a crucial role in pathogenesis and progression of RA in response to stimulation by inflammatory cytokines (Table 1). Abnormal activation of JAK/STAT pathway leads to increased MMP gene expression and cell resistance to apoptosis. JAKs family (JAK1, JAK2, JAK3, TYK2) are nonreceptor protein tyrosine kinases that are phosphorylated after the inflammatory cytokines, mainly IL-6, bind to its receptors. JAKs activation leads to recruitment of STAT. The STAT family includes 7 members (STAT1, STAT2, STAT3, STAT4, STAT5a, STAT5b, STAT6) that are inactive in the cytosol under normal physiological state. Recruited STAT molecules are phosphorylated through the JAKs to form dimers. The dimers enter the nucleus, bind to DNA, and regulate the transcription of target genes. STAT3 functions as a transcription factor in various cellular processes, i.e., initiating an immune response, cell proliferation, migration, differentiation, and apoptosis [20, 132–134]. Both the hyperactivation and inactivation of the STAT3 can be associated with the occurrence of autoimmune diseases [135, 136]. In our review, we focused on STAT3 which plays a key role in the differentiation of Tfh, Th17, and Th22 and is related to the cytokines we describe.

**Table 1:** Janus kinase/signal transducer and activator of transcription (JAK/STAT) signaling pathway members. Cytokines, cytokine receptors, and phosphorylated JAKs associated with the specific subunits and phosphorylated STAT3

Interleukin	IL-6	IL-21	IL-22	IL-23
Receptor	IL-6R	IL-21R	IL-22R	IL-23R
JAK	JAK1 (gp130) JAK2 (gp130) TYK2 (gp130) [20]	JAK1 (IL-21R) JAK3 (γ-chain) [151]	JAK1 (IL-22R1) TYK2 (IL-10R2) [152]	JAK2 (IL-23R) TYK2 (IL-12Rβ1) [153]
STAT	STAT1 STAT3	STAT1 STAT3 STAT5	STAT1 STAT3 STAT5	STAT3 STAT5

In a study of mice with CIA, it was shown that inhibitor COX2, meloxicam, can inhibit STAT3 activation and act as an arthritis inhibitor blocking the formation of osteoclasts, thereby eradicating cartilage erosion [137]. Meloxicam is a nonsteroidal anti-inflammatory drug (NSAID), recommended at the lowest effective doses for patients with RA, JIA, and osteoarthritis [138]. Researchers investigated the effects of the alkaloid ellipticine isolated from the plant *Apocynaceae* on RA-FLS. Ellipticine inhibited STAT3 phosphorylation and induced RA-FLS apoptosis, suggesting potential benefit in the future treatment of RA [139]. The STAT3 pathway is involved in arthritis along with other cytokines and has been described earlier. It should be mentioned, however, that studies were also conducted, inter alia, on the hypoxic effects on STAT3-induced inflammatory pathways. The results showed that hypoxia induces activation, proliferation, and survival of RASFs cells and endothelial cells. It can also induce cytokines, chemokines, and MMPs [140]. A novel study revealed that IL-17 promotes FLSs proliferation and autophagy via STAT3 activation [141]. This result suggests that targeted therapy in STAT3 can bring beneficial effects for patients with RA. Research showed that the JAK/STAT pathway, as well as the production of autoantibodies, can be inhibited by ruxolitinib in cells from patients with SLE [142]. SLE is a multisystemic autoimmune disease, characterized by the production of pathological amounts of numerous autoantibodies. In contrast to RA, patients with SLE can develop a very serious organ (kidney failure, blood clotting problem, seizures) and skin complications [143]. Increased levels of STAT3 and Th17 cells were detected in both SLE and RA. Targeting the STAT3/Th17/IL-17 axis may have therapeutic benefits. In addition, the inhibitory drugs for SLE, tofacitinib and baricitinib, have been approved by European Medicines Agency for the treatment of RA [142]. Results published in 2019 presented final effects of tofacitinib safety and effectiveness in RA therapy [144].

Research published in 2016 showed the effect of tyrosine phosphatase (PTEN) on inhibition of STAT3. Overexpression of PTEN affected the inhibition of CIA development and reduced T cell activation. Reduced p53 expression in RA patients affected STAT3 activation and increased Th17 proliferation, since p53 acts as a balance controller between Th17 and Treg via STAT3. Thus, PTEN, by decreasing STAT3 activity, can inhibit disease progression and be a therapeutic factor [145]. Studies conducted by Deng et al. [146] showed that CD4+ Tfh cells support the production of autoantibodies and participate in the promotion of inflammation. These studies showed that there is a relationship between elevated IL-6, phosphorylated STAT3, and Tfh cell concentration and suggested targeting this immunoregulatory axis in future RA studies. Study focusing on Tfh cells showed that there is an imbalance between Tfh and Treg cells which can be crucial for RA and serve as a therapy target. In addition, they showed that increased IL-6 concentration activates the STAT3 pathway that can promote this Tfh and Treg cell imbalance by the fact that STAT3 is a regulator of Tfh cell differentiation [136]. STAT3 which lacks RORγt induction is another transcription factor leading to cell differentiation towards Th17 [147].

Studies have also shown the effect of curculigoside isolated from *C. rhizoma*. *In vivo* and *in vitro* studies after collagen stimulation have shown a reduction in secretion of TNF-α, IL-1β, IL-6, and IL-17A. Curculigoside also reduced the expression level of JAK1, JAK3, and STAT3 in cells stimulated with TNF-α, while increasing the expression level of NF-κB. These results may indicate a large contribution of the JAK/STAT/NF-κB pathway in signal transduction, and curculigoside may affect regulation of this pathway and therapeutic effect in RA [148].

As noted, STAT3 is one of the main factors that orchestrate multiple inflammatory pathways and may play an important role in inhibition of interleukins, regulating the expression of MMPs genes and apoptosis of T cells [149]. JAK/STAT signaling affects numerous cytokines; therefore, the further development of drugs could be directed towards the development of inhibitors of this pathway, especially since tofacitinib has been used successfully in the treatment of rheumatic diseases [150].

## Conclusions

Autoimmune rheumatic diseases are a group of diseases whose etiology is not fully understood. As shown in this paper, many studies are conducted on arthritis affecting both adults and children. Research focuses on discovering new therapeutic targets for autoimmune diseases, while introducing anti-inflammatory drugs. They can be both monoclonal antibodies and fusion proteins, but also other therapeutic agents that effectively inhibit inflammatory pathways. The anti-inflammatory drugs discussed in this article are summarized in Table 2.

**Table 2:** Anti-inflammatory drugs, treatment mechanism, and side effects after treatment in autoimmune arthritis

Group	Drug	Mechanism	Side effects after treatment	Approved for treatment in arthritis
Monoclonal antibody	Adalimumab	Fully human anti-TNF-α monoclonal antibody [34]	Upper respiratory track infection, injection site reaction, sinusitis, pharyngitis/tonsillitis, ear infection [156]	+
	Bimekizumab	Humanized monoclonal IgG1 antibody, dual inhibitor of IL-17A and IL-17F [83]	Nasopharyngitis, headache, oropharyngeal pain, medical device (ECG) site reaction [82]	-
	Brodalumab	Fully human anti-IL-17A receptor monoclonal antibody [84]	Nasopharyngitis, upper respiratory track infection, urinary tract infection, injection site pain, hypertension [84]	- (psoriasis treatment)
	Guselkumab	Fully human anti-p19 IL-23 subunit monoclonal antibody [128]	Nasopharyngitis, upper respiratory track infection, alanine aminotransferase increased, aspartate aminotransferase increased [128]	+
	Ixekizumab	Recombinant, humanized anti-IL-17A monoclonal antibody with high affinity to G4κ [79]	Nasopharyngitis, upper respiratory tract infection, injection-site erythema, bronchitis [79]	- (psoriasis treatment)
	Risankizumab	Humanized anti-p19 IL-23 subunit monoclonal antibody [129]	Nasopharyngitis, arthralgia, headache [157]	- (psoriasis treatment)
	Sarilumab	Human anti-IL-6 receptor monoclonal antibody [34]	Upper respiratory track infections, urinary tract infections, neutropenia, injection site erythema [34]	+
	Secukinumab	Fully human anti-IL-17A monoclonal antibody [76]	Nasopharyngitis, upper respiratory tract infection , hypertension, diarrhea, influenza, candida infection	- (psoriasis treatment)
	Sirukumab	Fully human anti-IL-6 monoclonal antibody [41]	Nasopharyngitis, upper respiratory tract infection, urinary tract infection , injection-site reactions, hypertension [41]	-
	Tocilizumab	Humanized anti-IL-6 receptor, genetically-engineered monoclonal antibody [158]	Nasopharyngitis, gastrointestinal perforations, hypersensitivity [33], skin and subcutaneous disorders [32]	+
	Ustekinumab	Fully human anti-IgG1κ monoclonal antibody, binds to the p40 subunit common to IL-12 and IL-23 [126]	Nasopharyngitis, urinary tract infection, injection site reactions, worsening of RA [159]	- (psoriasis treatment)
JAK/STAT pathway inhibitors	Baricitinib	Inhibitor of Janus kinases 1 and 2 [160]	Nasopharyngitis, upper respiratory tract infection, urinary tract infection, gastrointestinal disorders [161]	+
	Leflunomide	Inhibitor of pyrimidine biosynthesis and tyrosine phosphorylation, suppressor of B cell antibody response [106]	Hepatic enzyme increase [162]	+
	Meloxicam	Inhibitor of COX2 and STAT3 [137]	Urinary tract infection, gastrointestinal disorders, cardiovascular disorders [138]	+
	Tofacitinib	Inhibitor of Janus kinases 1, 2 and 3 [160]	Nasopharyngitis, upper respiratory tract infection, urinary tract infection, gastrointestinal disorders, bronchitis [144]	+
Others	Enalapril	Angiotensin II type 1 receptor (AT_1_R) antagonist; inhibitor of IL-1β expression [114]	No side effects [115]	-
	Etanercept	Recombinant fusion protein against TNF-α [163]	Nasopharyngitis, upper respiratory tract infection, gastrointestinal symptoms [29]	+
	Ibudilast	A non-selective phosphodiesterase (PDE) inhibitor, mainly PDE4 [62]	Gastrointestinal symptoms, depression, insomnia, fatigue (in progressive multiple sclerosis) [164]	-
	Losartan	Angiotensin II type 1 receptor (AT_1_R) antagonist; inhibitor of pro-inflammatory cytokine IL-6, IL-17, IL-22 and IFN-γ [114, 165]	Nervous system disorders, cardiac disorders, dizziness/presyncope, musculoskeletal and connective tissue disorders (in low-grade chronic inflammation) [166]	-
	Methotrexate	Inducer of T cells apoptosis and inhibitor of cell proliferation, associated with adenosine-mediated mechanism [167]	Muscle spasms, nausea, diarrhea, headache [168]	+
	Sulfasalazine	Inhibitor of NF-κB activation; inhibitor of pro-inflammatory cytokine and MMPs production, suppressor of cell proliferatin [73]	Gastrointestinal symptoms, pneumonia, breast cancer, bronchitis [169]	+
	Sulforaphane	Regulator phase II enzymes and apoptosis [64]	Bloating (high glucosinolate group) [71]	-

Further development may also be focused on the detection of effective and safe second- or third-line drugs that will provide greater safety and reduce infection and other adverse events after administration of the first-line drug or lack of its effectiveness [154]. Patient drug resistance is also a challenge for researchers, so it is important to focus on developing e.g. effective dose and dosing schedules. It should also be checked whether drugs have the same effect on different subgroups of RA by analyzing RA+/RA- or ACPA+/ACPA- patients. In recent years, gene therapy methods used to treat RA have also aroused interest. The conducted research offers hope for the development of new, safe, and effective methods of treatment that would allow systemic immune suppression. Gene therapy would allow the inhibition of pro-inflammatory cytokines and the overproduction of anti-inflammatory cytokines, inhibiting or promoting apoptosis and inhibiting angiogenesis. The elimination of many barriers, i.e., the selection of an appropriate vector or the choice of the route of administration, would have a decisive impact on the effectiveness of the treatment [155].
